# Transferrin conjugation confers mucosal molecular targeting to a model HIV-1 trimeric gp140 vaccine antigen^☆^

**DOI:** 10.1016/j.jconrel.2011.11.009

**Published:** 2012-03-10

**Authors:** J.F.S. Mann, D. Stieh, K. Klein, D.S. Miranda de Stegmann, M.P. Cranage, R.J. Shattock, P.F. McKay

**Affiliations:** a Centre for Infection and Immunity, Division of Clinical Sciences, St. George's, University of London, London, SW17 0RE, United Kingdom; b Imperial College London, Department of Infectious Diseases, Division of Medicine, Norfolk Place, London, W2 1PG, United Kingdom

**Keywords:** Vaccine delivery, Targeting, Mucosal, Transcytosis, Immunogenicity

## Abstract

The generation of effective immune responses by mucosal vaccination without the use of inflammatory adjuvants, that compromise the epithelial barrier and recruit new cellular targets, is a key goal of vaccines designed to protect against sexually acquired pathogens. In the present study we use a model HIV antigen (CN54gp140) conjugated to transferrin (Tf) and evaluate the ability of the natural transferrin receptor CD71 to modulate immunity. We show that the conjugated transferrin retained high affinity for its receptor and that the conjugate was specifically transported across an epithelial barrier, co-localizing with MHC Class II^+^ cells in the sub-mucosal stroma. Vaccination studies in mice revealed that the Tf-gp140 conjugate elicited high titres of CN54gp140-specific serum antibodies, equivalent to a systemic vaccination, when conjugate was applied topically to the nasal mucosae whereas gp140 alone was poorly immunogenic. Moreover, the Tf-gp140 conjugate elicited both IgG and IgA responses and significantly higher gp140-specific IgA titre in the female genital tract than unconjugated antigen. These responses were achieved after mucosal application of the conjugated protein alone, in the absence of any pro-inflammatory adjuvant and suggest a potentially useful and novel molecular targeting approach, delivering a vaccine cargo to directly elicit or enhance pathogen-specific mucosal immunity.

## Graphical abstract

A stepwise illustration of the utilization of the highly active biological process of transcytosis for mucosal vaccine targeting and enhanced immunogenicity.

**Figure:**
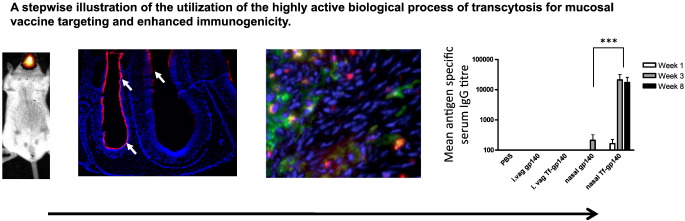

## 1 Introduction

Mucosal epithelia and associated lymphoid tissues are the primary route of entry for a large number of infectious pathogens, including sexually transmitted infections that have a high and increasing incidence. Effective vaccination strategies should establish immunity at these portals of entry, the exposed mucosa and local draining lymph nodes. Antibodies present at mucosal surfaces have the potential to completely prevent overt infection by enhancing the protection provided by the mucosal physical barrier, but to stimulate such a response it is likely that vaccination modalities will require a mucosal component that can enlist and direct responses to “home” to mucosal tissue, while maintaining the integrity of the barrier mucosae. Most vaccination regimes, however, use potent adjuvants that enhance the apparent “danger” of a vaccine antigen, by stimulation of several families of pattern recognition receptors (PRRs) and/or direct recruitment of immune responsive cells, leading to local inflammation and disruption of mucosal epithelial integrity [1,2]. The mucosa is a highly structured, complex tissue with distinct areas that have dense aggregations of T, B, dendritic cells and macrophages as well as areas virtually devoid of immune cells [3]. The immune cells can be either normally resident or transient sentinels that survey mucosal tissue for incoming antigen or danger signals and all cells within the mucosae, including epithelial and stromal cells, exhibit a high degree of cross-talk, two-way interactions that can often have significant down-stream functional effects upon the elicitation of immune responses to a vaccine antigen [4–6]. The cell type within the sub-mucosae and mucosal lamina propria likely to be central to elicitation of both mucosal and systemic immune responses is the professional antigen-presenting dendritic cell and we set out to deliver antigen to these target cells [3,6]. We hypothesized that increasing the amount of vaccine antigen transported across the epithelial barrier will augment dendritic cell elicited immune responses and utilized the natural transcytotic capacity of the transferrin receptor CD71 to achieve this goal.

Our overall aim was to generate immune responses within the vaginal mucosae and it has previously been shown that the female genital tract has some immune inductive potential. For example, intravaginal vaccination studies utilizing Toll-like receptor (TLR) agonists and CD4^+^ T helper epitopes [7] or powerful adjuvants such as cholera toxin [8] have demonstrated that immunization at this mucosal surface is possible but responses elicited to unadjuvanted vaccine antigens, where vaginal epithelial barrier integrity is maintained, have been modest [9,10]. This may be due to a number of reasons, distinct from the recruitment and activation of immune responsive cells, such as the inability of vaccinating antigens to traverse the type II stratified squamous epithelial barrier characteristic of the lower female reproductive tract [11], the possibility that the female reproductive tract is directed more towards an induced state of tolerance rather than inflammation [12], vaginal leakage of the applied antigen [13] or sequestration and elimination of the antigen by locally produced mucus and proteases [14,15]. For human immunodeficiency virus (HIV) there is the additional concern that vaccine/adjuvant-induced local inflammation may fuel infection through the recruitment of target cells as suggested by a recent clinical trial. The MRKAd5 HIV-1 gag/pol/nef vaccine appeared to result in a higher HIV-1 incidence in the vaccine-treated cohort than the placebo-treated cohort, particularly amongst uncircumcised men who had a pre-existing Ad5 immunity, and who may have experienced higher local inflammation than control subjects [16]. Therefore, the challenge facing any vaginal mucosal vaccine is how to overcome these potential pitfalls.

We have therefore focused on an intranasal mucosal vaccine delivery system with low inflammatory potential that is capable of delivering an immunizing cargo and eliciting systemic and intravaginal immune responses. We have investigated a molecular targeting based approach to deliver a model vaccine cargo to mucosal tissues with the aim of increasing vaccine antigen bio-availability within the mucosal lamina propria and enhancing immune responses elicited by cells resident or transiently present in mucosal compartments. We used transferrin as the targeting component conjugated to a trimeric HIV gp140 model vaccine antigen cargo via biotin–streptavidin linkage (Tf-gp140). Transferrin targets the highly efficient transcytotic and recycling transferrin receptor (CD71) that is expressed on both nasal and vaginal mucosal epithelium, is actively transcytosed and has been successfully utilized as a delivery system for drug conjugates, when associated with microparticles or emulsion formulations, for the delivery of anti-cancer agents and in gene therapy [17]. Importantly, transferrin has been utilized as a trans-mucosal delivery modality for oral insulin preparations in a rat model of diabetes, suggesting that the CD71-transferrin transcytotic route has a realistic potential as an effective mucosal vaccine delivery strategy [18]. We sought to stringently test this hypothesis through linkage of transferrin to a large, stable trimeric Clade C HIV gp140 model antigen that was topically applied to mucosal epithelium that expressed apical to basolateral cycling CD71 molecules.

We report here the ability to generate systemic and vaginal antibody responses after nasal but not vaginal immunization in the absence of an adjuvant. Nasal mucosal immunization alone with the Tf-gp140 conjugate elicited comparable serum and mucosal titres to those obtained after prior subcutaneous priming followed by nasal mucosal boosting with unconjugated gp140. Interestingly however, nasal immunization alone with Tf-gp140 induced a striking IgG1 (Th2) biased serum response and a significant augmentation in anti-gp140 mucosal IgA response in cervico-vaginal fluid. Thus, a mucosally-applied and receptor-targeted approach can generate high titre antibody-specific responses to a large vaccine antigen conjugate in both the systemic and mucosal compartments highlighting the utility of Tf-gp140 as an effective means to generate vaginal antigen-specific antibody responses while unconjugated gp140 alone was ineffective.

## 2 Materials and methods

### 2.1 Recombinant trimeric HIV-1 gp140

A clade C HIV-1 envelope clone, designated p97CN54, was obtained from an HIV infected Chinese patient [19,20] and made available by Prof. H. Wolf and Prof. R. Wagner (University of Regensburg, Germany). Trimeric gp140 clade C envelope (gp120 plus the external domain (ED) of gp41), designated CN54gp140, was produced as a recombinant product in CHO cells (S. Jeffs—personal communication), and the protein manufactured to GMP specification by Polymun Scientific (Vienna, Austria). The fidelity of the product was confirmed by mass spectrometric analysis of tryptic fragments by the Medical Biomics Centre at St. George's, University of London. The trimeric product was stable, even when kept at room temperature (D. Katinger—personal communication) and has previously been reported to be immunogenic [21,22].

### 2.2 Transferrin-gp140 chemical conjugation

gp140 antigen was reacted with streptavidin in a 1:1 molar ratio using a Lightning-Link™ conjugation kit (Innova Biosciences, UK). Briefly, CN54gp140 was modified using the LL-modifier and mixed gently. Modified CN54gp140 was then added to the streptavidin and the reaction was incubated at room temperature for 3 hours before the reaction was stopped using the LL-quench. The modified protein was incubated for a further 30 min to ensure that the reaction had terminated. A relevant volume of biotinylated transferrin (Sigma, UK) at 5 mg/ml was then added to the gp140 streptavidin and the reaction was incubated for 1 hour.

### 2.3 Malvern zeta sizer analysis of conjugate

Conjugate size and particle distribution were measured using dynamic light scattering with a Malvern Zeta-Sizer (Malvern Instruments Ltd., UK) at 25 °C. Briefly, 10 μg protein was diluted into 300 μl PBS and placed into an analysis cuvette (Sigma-Aldrich Ltd., UK). The intensity of laser light scattered by the sample preparations was measured at 173° to the incident beam. The data were analyzed using proprietary Malvern software, DTS (Nano Version 5.0), supplied with the machine. The size distribution and the polydispersity were measured using non-invasive back scatter (NIBS).

### 2.4 Receptor–ligand binding affinity

The binding properties of the Tf-gp140 conjugate to transferrin receptor (CD71) were assessed using a Rapid 4 acoustic biosensor (TTP LabTech, UK). CD71 (Europa Bioproducts Ltd., UK) was immobilized on a quartz crystal chip (AccuBio, UK), and then either apo-transferrin, holo-transferrin or the Tf-gp140 conjugate was allowed to flow over the bound ligand. The binding of the different forms of transferrin or transferrin conjugate to the chip-bound CD71 effects a change in bound mass, which is detected as a difference in resonant frequency of the quartz cassette, allowing the determination of the binding kinetics between the transferrin alone or Tf-gp140 conjugate and the CD71, according to the Langmuir model.

### 2.5 Primary endocervical epithelium isolation, flow cytometric analysis and in vitro transwell studies

Cervical mucosal tissue was collected from pre-menopausal women undergoing therapeutic hysterectomies at St. George's, and Kingston Hospitals (London, United Kingdom); the tissue was dissected, digested with dispase II and primary endocervical cultures were established. These cells were analyzed by flow cytometry and used in transwell experiments (supplementary methods).

### 2.6 Mice, immunization and sampling

Female BALB/c mice (Harlan, UK), 6–8 weeks old, were placed into groups (*n* = 8) and housed in a fully acclimatized room. All animals were handled and procedures were performed in accordance with the UK Home Office Animals (Scientific Procedures) Act 1986. One week prior to the commencement of the study and every 4 weeks thereafter, mice receiving vaginal immunizations were treated with 2 mg of medroxy-progesterone (Pharmacia, UK) administered subcutaneously to synchronize the menstrual cycle and thin the vaginal epithelium. Food and water were supplied *ad libitum*.

Mice were immunized at 2 weekly intervals with three intravaginal or intranasal mucosal applications of 50 μg gp140 or Tf-gp140 conjugate. Alternatively, the mice were exposed to the gp140 antigen before mucosal vaccination by a single subcutaneous injection of 10 μg gp140 followed by the three, two weekly interval, 50 μg intravaginal or intranasal immunizations with gp140 or Tf-gp140. A control group received a single subcutaneous injection of 10 μg gp140. Tail bleeds were collected weekly without anti-coagulant and centrifuged in a Heraeus Biofuge Pico (Fisher, UK) at 1000*g* for 10 min. The serum was harvested and transferred into fresh 0.5 ml micro-centrifuge tubes (Starlabs, UK), and stored at − 20 °C until antibody titres were determined by indirect ELISA. Vaginal lavage was carried out weekly using three 25 μl washes/mouse with PBS that were subsequently pooled. Lavage samples were incubated for 30 min with 4 μl of 25× stock solution protease inhibitor (Roche Diagnostics, Germany) before centrifuging at 1000*g* for 10 min. The fluid supernatant from these treated samples was then transferred into a fresh 0.5 ml micro-centrifuge tube, and stored at − 20 °C until antibody titres were determined by indirect ELISA.

### 2.7 In vivo fluorescence

Real time bio-imaging of topically applied fluorescently-labeled transferrin was performed using a multispectral Carestream In Vivo FX Pro system (USA). Briefly, a 100 μg dose of Tf-Alexa 647 nm was applied in a 15 μl volume to the vaginal or nasal mucosa of anesthetized female BALB/c mice. Imaging was carried out immediately after application of Tf-Alexa and at various time points dependent on the tissue of interest. Photonic emissions were captured after a 15 second exposure with a 670 nm filter and images were acquired and analyzed using Carestream software (USA).

### 2.8 ELISA

Samples from primary cell cultures or immunized mice were variously analyzed using an anti-human transferrin, a CN54gp140 antigen-specific and an anti-CN54 antibody ELISA. Full details of the ELISA methods used are included in the supplementary method section.

### 2.9 Immunohistochemistry

To visualize the ability of transferrin or the Tf-gp140 conjugate to translocate into the submucosal environment from an external luminal compartment, vaginal or nasal tissue was removed from treated animals, and then embedded in OCT Cryomatrix (RA Lamb, USA) and flash frozen in liquid nitrogen. Endocervical tissue from patients undergoing planned therapeutic hysterectomy (local Research Ethics Committee approval was obtained) were cut into 3 mm^3^ samples and placed into 10% formaldehyde overnight at 4 °C. Tissue samples were loaded into histocasettes (Fisher, UK) and paraffin embedded overnight. Sections were cut from these prepared tissues and stained as described in the supplementary methods section.

### 2.10 Statistical analysis

Statistical analysis of the data was carried out by the Mann–Whitney rank-sum test using Prism (GraphPad Software, Inc., USA).

## 3 Results

### 3.1 Transferrin-CN54gp140 conjugate and native transferrin have similar CD71 binding affinities

**Fig. 1:**
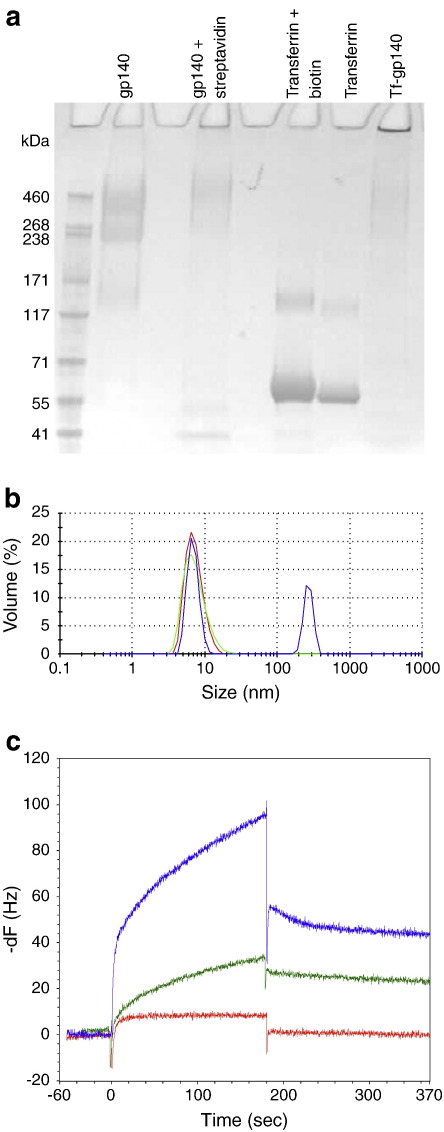
Formation, physical and functional analysis of Tf-gp140 conjugate. a) Tris-acetate gel electrophoresis of gp140, streptavidinated gp140, transferrin, biotinylated transferrin and the conjugate formed by combination of the streptavidin- and biotin-labeled components. Optimal conjugate formed at a ratio of 4:1 biotinylated transferrin:gp140-streptavidin (Tf-gp140; Lane 8). b) Size distribution curves normalized for % volume for transferrin (), gp140 () and the Tf-gp140 conjugate () obtained by dynamic light scattering at 25 °C using a Malvern Zeta-sizer. The intensity of the laser light scattered by the sample preparations was detected at 173° to the incident beam. Data were analyzed using Malvern DTS (Nano Version 5.0) software. c) Apo-transferrin (), holo-transferrin () and Tf-gp140 conjugate () binding and dissociating with immobilized transferrin receptor (CD71) were assessed using an acoustic biosensor.

To utilize the highly efficient transcytotic capacity of the CD71 transferrin receptor, biotinylated transferrin was conjugated to streptavidinated recombinant trimeric HIV CN54gp140. The addition of streptavidin increased the apparent relative molecular mass of gp140 as did the addition of biotin to transferrin (Fig. 1a). We found that a 4:1 molar ratio of transferrin–biotin to CN54gp140-streptavidin was necessary to combine all reactants efficiently. This resulted in a conjugate that just entered a 7–10% gel due to its large mass (Fig. 1a, Tf-gp140). Zeta–Sizer analysis of this conjugate revealed that it had a more poly-dispersed profile than either component alone and contained particle sizes ranging from 200 to 400 nm in diameter (Fig. 1b) in addition to smaller species. Next, the ability of the conjugate to bind to the transferrin receptor, CD71, was determined by resonant acoustic profiling (Fig. 1c). The CD71 molecule was covalently bound to the sensor chip and the binding of the three different transferrin moieties (holo-transferrin, apo-transferrin or Tf-gp140) was measured. The association and dissociation rates for transferrin or the Tf-gp140 conjugate (normalized for transferrin concentration) were used to calculate affinity for chip-bound CD71. The multi-molecular Tf-gp140 conjugate complex retained specific affinity for CD71 that was approximately 2-fold greater than transferrin alone, with a 4.45 × 10^− 8^ M affinity of Tf-gp140 and a 1.02 × 10^− 7^ M affinity of transferrin for the immobilized CD71 (Fig. 1c). Apo-transferrin showed no specific binding to the CD71 molecule.

### 3.2 The Tf-gp140 conjugate is actively and efficiently transcytosed across human mucosal primary columnar epithelium in vitro

**Fig. 2:**
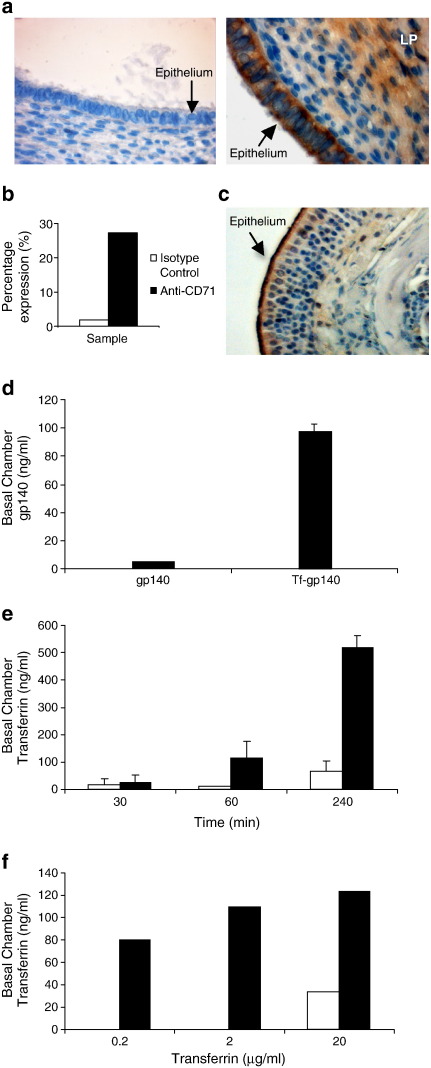
Human transferrin transcytosis is an active process that can transport an attached vaccine antigen. a) Expression of CD71 on human endocervical columnar epithelium. Sections were stained for human CD71 (right panel) or an isotype control (left panel). b) Primary epithelial cells isolated from endocervical tissue were surface stained with isotype-matched control or CD71-specific fluorochrome labeled antibodies and expression analyzed by flow cytometry. c) Expression of murine CD71 on intranasal epithelium. d) Transferrin can efficiently transport a large vaccine antigen. A specific ELISA quantified gp140 in the basal transwell chamber 24 hours after application of either unconjugated gp140 (50 μg) or the Tf-gp140 (50 μg) conjugate to the apical chamber. The resistance across the transwell chambers was maintained throughout all experiments. e) Human transferrin can traverse an intact human primary cervical epithelium transwell system. Human transferrin (500 μl of 1 mg/ml) was added to the apical chamber of a transwell culture dish containing confluent human cervical epithelial cells and transferrin concentrations in the basolateral chamber determined using a human transferrin ELISA after 30, 60 and 240 min in the presence (□) or absence (■) of monensin. f) This specific transferrin transport occurs even at low apical chamber transferrin concentrations, samples removed form basal chamber at 24 hours.

Confluent monolayers of primary columnar epithelial cells derived from human endocervical tissue cultured directly ex vivo were used to assess the ability of transferrin to transport a bound cargo. The expression of CD71 on endocervical and murine intranasal surface epithelia was confirmed by immunohistochemistry (Fig. 2a and c). Surface CD71 expression was further analyzed by flow cytometry on the primary cultured epithelial cells immediately prior to use in the transwell system and showed that CD71 expression was retained after isolation and short-term culture (Fig. 2b).

The ability of transferrin to specifically mediate the transport of conjugated gp140 across the epithelial layer was evaluated. Tf-gp140 or unconjugated gp140 was placed into the apical chamber of the transwell system, in the presence or absence of the protein transport inhibitor monensin, having confirmed the formation of intercellular tight junctions by resistance across the well. After 24 hours the Tf-gp140 transversed the confluent epithelium 29.3 times more efficiently than unconjugated gp140 and this transport was abolished in the presence of monensin (Fig. 2d). Next, human transferrin was applied to the apical chamber of a transwell system containing a confluent layer of primary human endocervical epithelial cells. Transferrin was detected in the basolateral chamber within 30 min and continued to accumulate, demonstrating a time-dependent transport (Fig. 2e). This transport was, as expected, also arrested using the intracellular protein transport inhibitor monensin indicating that the transport was an active process (Fig. 2e). Active transport of transferrin across the primary epithelia also occurred at low transferrin concentrations (Fig. 2f). Importantly, confluency and high resistance were maintained throughout the course of the experiments.

### 3.3 Transferrin or Tf-gp140 traverse intact mucosal epithelium in vivo and co-localize with submucosal APCs

**Fig. 3:**
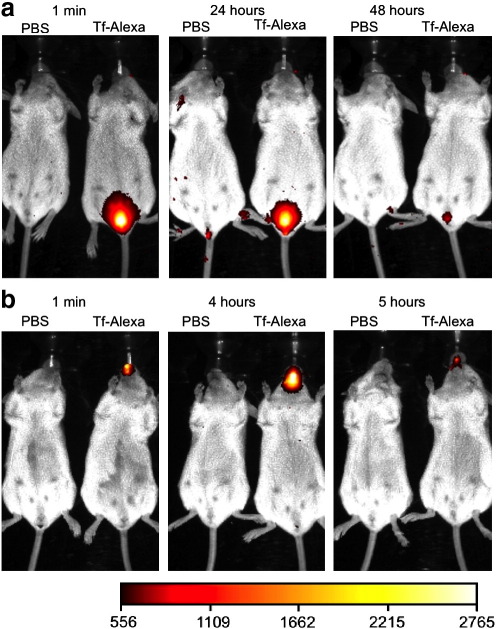
Kinetics of in vivo Tf-Alexa fluorescence after topical administration to vaginal and nasal mucosa. 100 μg Tf-Alexa in a 15 μl volume was applied topically into the mouse nasal cavity or the vaginal vault. a) Fluorescent signal acquired with a 1  second exposure through a 670 nm filter after vaginal application to the right-hand mouse in each panel. Images were taken at various times and the signal captured immediately after application and at 24 and 48 hours is shown. b) Signal acquired after topical application into the nasal cavity. Images were taken immediately after application and at 4 and 5 hours. No fluorescence was seen after 5 hours in any of a number (*n* > 3) of nasal applications. Animals were maintained under anesthetic during imaging but were fully awake and active between each image capture. Image sample times are shown above each panel while the scale bar represents fluorescence light intensity.

Having determined that human transferrin can cross primary human cervical epithelium in vitro, we next sought to visualize and then assess the ingress of transferrin using an in vivo model. We first examined the residence time of topically applied human transferrin within the murine nasal cavity or vaginal vault (Fig. 3a–b). We noted from in vivo imaging analysis that fluorescently labeled human transferrin was detectable within the vaginal vault for up to 48 hours (Fig. 3a) while the same quantity and volume of fluorescent transferrin was only observed in the nasal cavity for up to 5 hours (Fig. 3b).

**Fig. 4:**
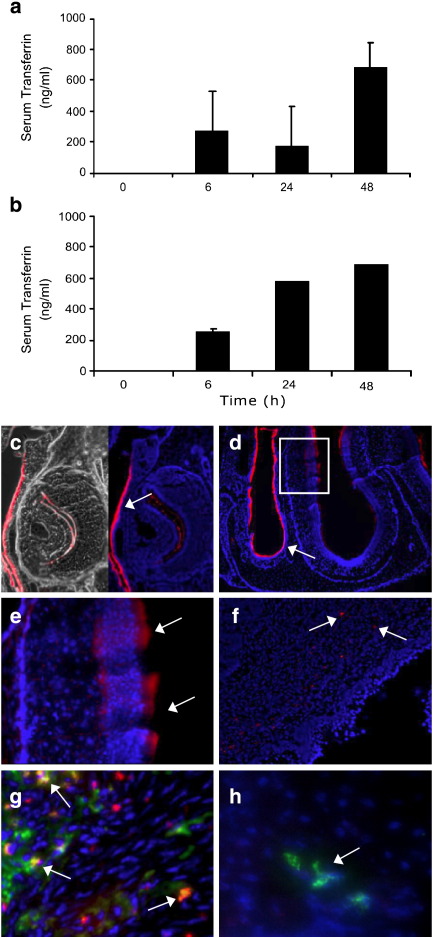
Transferrin crosses mucosal epithelium in vivo, localizes to MHC Class II+ cells and can transport a vaccine antigen to APC in the underlying stroma. a–b) Human transferrin was quantified by specific ELISA in murine serum samples taken 6, 24 and 48 hours after application of human transferrin (20 μl of 1 mg/ml) into the vaginal vault (a) or topically to the nasal mucosae (b). c) Tf-Alexa 647 applied intranasally in mice was visualized in epithelium and within sub-epithelial stromal tissue (lamina propria). Fluorescent signal is shown in phase contrast and with 4′-6-diamidino-2-phenylindole (DAPI)-stained nuclei. d) Tf-Alexa 647 signal in nasal turbinate epithelia. e) High power magnification (× 400) of white box highlighted in (d) showing progress of fluorescent Tf through epithelia and accumulating within cells in stroma. f) Tf-Alexa 647 applied intravaginally in mice was visualized within sub-epithelial lamina propria 2 hours after application (arrows). Tf-Alexa 647 accumulated within cells enabling detection, as diffuse Tf-Alexa 647 cannot be seen. g) After intravaginal administration of Tf-Alexa 647 (red), signal was found to co-localize with a proportion of MHC Class II^+^ cells revealed by staining with a FITC labeled antibody (M5/114.15.2) (green) in the sub-epithelial stroma (co-localization—yellow, examples shown with arrows). h) After intravaginal administration of Tf-gp140 and staining with gp140-specific mAb 5F3 followed by a FITC labeled anti-Ig secondary, occasional cells with dendritic-like morphology were detected with distinctive punctate staining.

We then determined if human transferrin was able to functionally utilize the murine transferrin receptor. After application of transferrin to the vaginal vault or nasal mucosae of mice, human transferrin was detected by a human Tf-specific ELISA in serum as early as 6 hours after application (Fig. 4a–b). The concentration of human transferrin detected in the serum continued to increase for 48 hours after application by either route, with transferrin applied nasally accessing the murine circulatory system at a faster rate, resulting in higher levels being detected by 24 hours.

We then sought to track the progress of transferrin through the mucosal barrier and any initial interactions with immune cells in the sub-mucosal stroma. Here, we topically placed 100 μg of fluorochrome-labeled human transferrin (Tf-Alexa) into the vaginal or nasal lumens. After application tissues were extracted, processed and snap frozen and 6–8 μm sections were prepared. Tf-Alexa fluorescence was detected in epithelia lining the nostrils and nasal conchae (Fig. 4c–d). Under higher magnification, Tf-Alexa had clearly traversed the mucosal epithelia of the nasal passages and entered into the lamina propria, thus breaching the mucosal barrier and gaining access to areas well documented to have an abundance of immune inductive cells (Fig. 4e). Large amounts of Tf-Alexa can be seen throughout the epithelial layer with defined punctate staining within the tissue stroma. Examination of vaginal tissue, after topical application of Tf-Alexa, also revealed numerous punctate Tf-Alexa bodies being observed underneath the surface epithelium in the lamina propria indicating sufficient accumulation of the fluorochrome within cells to enable visualization (Fig. 4f). However, little Tf-Alexa could be detected within or at the epithelial surface. By staining for MHC class II in vaginal tissues of mice administered with Tf-Alexa, co-localization of Tf-Alexa and APCs could be observed demonstrating that the transferrin not only transverses the epithelial barrier, but can also access areas where immune inductive cells reside (Fig. 4g). To test proof of principle that Tf can carry a vaccine antigen through a mucosal epithelial barrier in vivo we applied 100 μg of the Tf-gp140 conjugate into the mouse vaginal vault, harvested the vagina after 2 hours and examined sections for the presence of gp140 protein using the gp140-specific 5F3 antibody. Staining confirmed that gp140 protein had crossed the mucosal epithelium and was present in the stromal lamina propria of the vaginal tissue, becoming concentrated in cells with a dendritic morphology (Fig. 4h). No gp140 protein was detectable in sections from mice that had received unconjugated protein. Taken together these results indicate that the capacity for transferrin to carry a vaccine cargo across an epithelial layer was applicable in vivo as had been demonstrated in vitro.

### 3.4 Tf-gp140 elicits high-titre serum gp140-specific antibody responses after intranasal but not intravaginal application

**Fig. 5:**
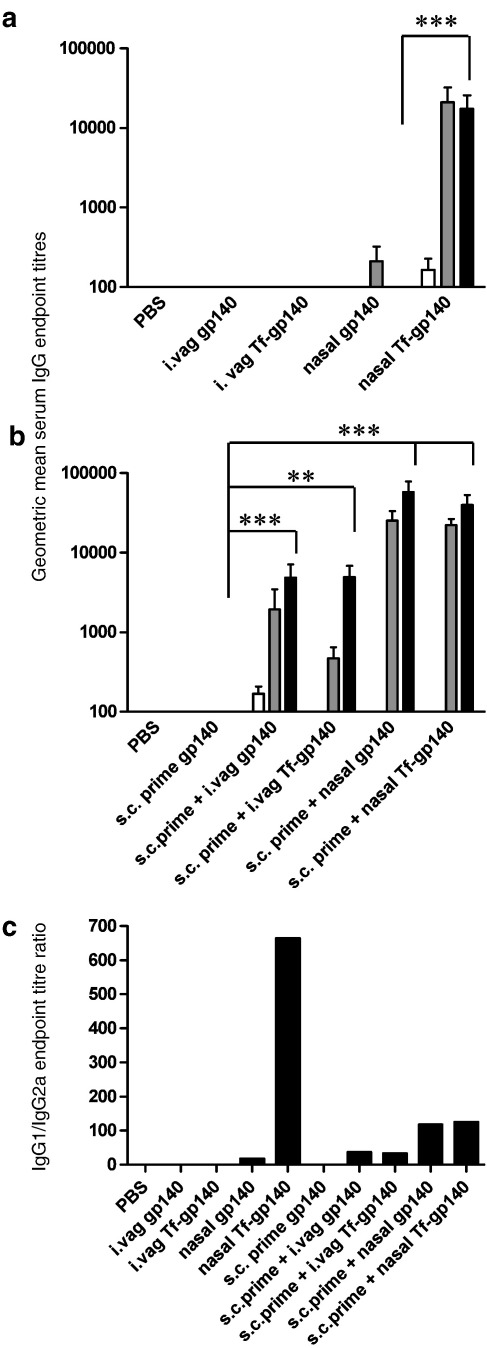
Application of Tf-gp140 to the nasal mucosae elicits potent antigen-specific serum antibody responses. Animals (n = 8) were vaccinated via the nasal or vaginal mucosal route three times at two-week intervals with or without a single prior subcutaneous prime. a) Serum IgG antibody geometric mean titres (GMT) assessed by ELISA at week 1 ( □ ), 3 () and 8 ( ■ ) following the initial mucosal application with either gp140 or the Tf-gp140 conjugate. b) Serum IgG antibody GMTs assessed by ELISA after a single subcutaneous prime with 10 μg unadjuvanted gp140 followed with three mucosal boosts. Timepoints shown are at week 1 ( □ ), 3 () and 8 ( ■ ) following the mucosal application with either gp140 or the Tf-gp140 conjugate. Values represent end point dilution titres ± S.D. of the group (n = 8) (***p = 0.0002 **p = 0.0019). c) Serum IgG1/IgG2a ratios were assessed at week 8 using the GMT of each isotype. Statistical analysis was carried out using a Mann Whitney rank-sum test with significance indicated by values *p* < 0.05.

To determine the immunogenic potential of the Tf-gp140 conjugate mice were immunized mucosally at two-week intervals with either 50 μg of gp140 or with 50 μg of Tf-gp140 conjugate, with or without a subcutaneous priming with gp140. Primed groups received a single subcutaneous injection of 10 μg of unadjuvanted gp140 protein 14 days before the first mucosal inoculation (Day 0). Animals that were vaccinated vaginally were pretreated with medroxyprogesterone to thin the highly stratified murine vaginal epithelium. Application of gp140, without adjuvants, to the nasal or vaginal vault elicited only minimal immunity (Fig. 5a).

Following intranasal administration of the Tf-gp140 conjugate, gp140-specific serum antibody responses were detected in 8 of 8 mice after a single application. Titres were strongly and significantly boosted with a second application, while the response to administration of gp140 alone was barely detectable (*p* = 0.0002) (Fig. 5a). A further intranasal vaccination did not increase titres in either group. The antibody titres generated by two mucosal applications of the Tf-gp140 conjugate were at least as high as levels achieved with prior systemic priming with gp140 and two mucosal boosts with either Tf-gp140 or the unconjugated gp140 antigen (*p* ≥ 0.05). In contrast to intranasal inoculation, application of Tf-gp140 to the vaginal mucosa did not elicit any serum gp140 antigen-specific responses, highlighting the difference between the immune inductive potential of these mucosal sites (Fig. 5a). However, in animals that were previously primed subcutaneously with gp140, serum antibody titres were boosted significantly by application of either gp140 (*p* = 0.0002) or Tf-gp140 (*p* = 0.0019) via the vaginal route (Fig. 5b). Interestingly, serum titres continued to rise after a third intravaginal vaccination but failed to reach the level obtained by intranasal vaccination. Likewise, the antibody titres in animals subcutaneously primed with unadjuvanted gp140 were boosted after nasal mucosal applications of unconjugated gp140; however, high titres were attained in this instance after only two mucosal immunizations (Fig. 5b). Crucially, serum anti-gp140 titres obtained after nasal mucosal administration of Tf-gp140 were not significantly enhanced by prior subcutaneous priming (*p* ≥ 0.05). Analysis of the isotype sub-class composition of the gp140 antigen-specific serum antibodies revealed that all animals that responded showed an IgG1 isotype bias, likely due to the natural Th2 skewing of BALB/c mice, but those animals that had been mucosally vaccinated with the Tf-gp140 conjugate exhibited a dramatic further enhancement to the existing IgG1 isotype bias, indicating that the Tf-gp140 conjugate may augment gp140-specific B cell differentiation (Fig. 5c).

### 3.5 Tf-gp140 elicits an IgA biased cervico-vaginal response following intranasal application

**Fig. 6:**
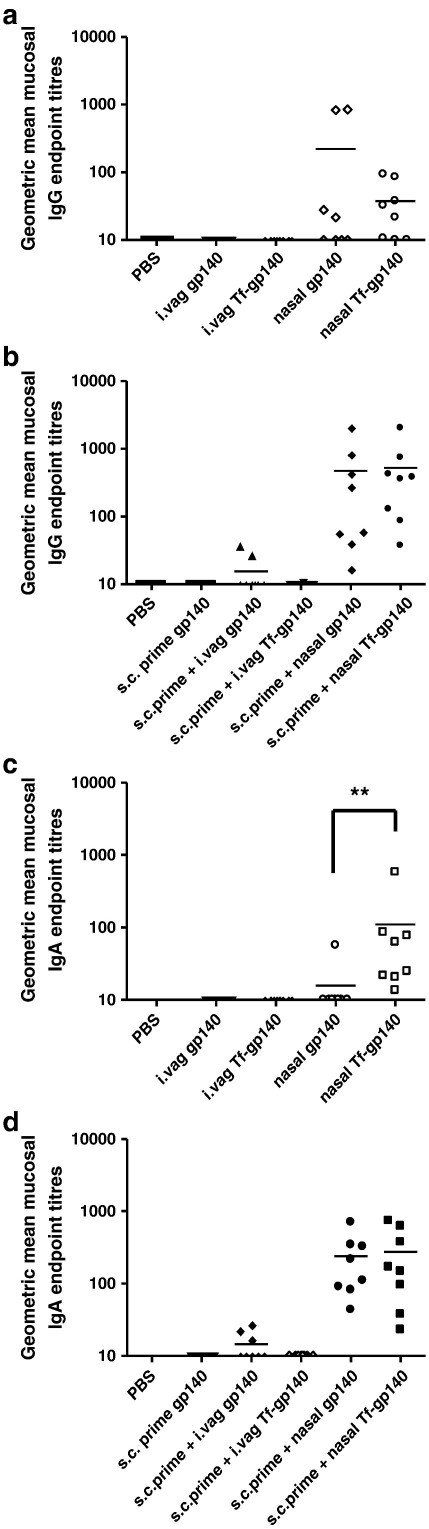
The Tf-gp140 conjugate significantly augments antigen-specific mucosal IgA production after application to the nasal mucosae. Antigen-specific mucosal IgG (a, b) and IgA (c, d) titres were measured in mucosal lavage samples from murine vaginal vaults 8 weeks following the initial mucosal application with either gp140 or the Tf-gp140 conjugate. The panels show the reciprocal endpoint dilutions, normalized for total Ig, of each individual mouse within a group. Open symbols (a, c) indicate that the mice received only mucosal applications of the formulations while closed symbols (b, d) indicate that the mice also received a subcutaneous injection with 10 μg unadjuvanted gp140 two weeks before any mucosal applications (**p = 0.0019). Statistical analysis was carried out using a Mann Whitney rank-sum test with significance indicated by values *p* < 0.05.

Intravaginal administration of either gp140 or Tf-gp140 in the absence of subcutaneous priming, failed to stimulate a detectable IgG or IgA response in cervico-vaginal lavage mirroring the lack of a serum response (Fig. 6a, c). However, intranasal administration of either gp140 or Tf-gp140 stimulated cervico-vaginal IgG antibody in 4 of 8 and 5 of 8 animals respectively with no statistical difference in the mean titre (*p* ≥ 0.05). Interestingly however, only 1 of 8 animals had cervico-vaginal IgA antibody after nasal gp140 application whereas 8 of 8 animals had cervico-vaginal IgA after nasal administration of Tf-gp140, with a statistically different GMT of 15.8 vs. 110.2 (*p* = 0.0019) (Fig. 6a, c). As observed for serum responses, subcutaneous priming with gp140 facilitated efficient nasal boosting with gp140, all animals responding with IgG and IgA responses and no statistically significant difference in GMT between gp140 or Tf-gp140 boosting (*p* ≥ 0.05) (Fig. 6b, d). Although the mean titre of cervico-vaginal IgA antibody was slightly higher if animals receiving nasal Tf-gp140 were primed subcutaneously (110.2 vs. 274.8 (*p* ≥ 0.05)), at least for IgA it was evident that conjugation of transferrin to antigen could overcome the absolute requirement for systemic immunization.

## 4 Discussion

Current thinking in vaccinology suggests that optimal generation and maintenance of mucosal immunity may require inoculation at, or within, mucosal tissues. However, vaccines comprised solely of proteins that are not themselves toxins or ligands for pattern recognition receptors are typically poorly immunogenic when administered systemically and are virtually immunologically invisible when applied topically to mucosal membranes [10,23,24]. Therefore to elicit potent and long-lasting immunity, whether mucosal or systemic, it is clear that the majority of potential protein vaccine antigens must be adjuvanted or “immunologically assisted” by pro-inflammatory biological or biochemical agents. Such adjuvants will result in an inflammatory response that can dramatically enhance a systemic immunization and elicit vaccine-specific immunity without significant damage to the recipient and are already widely accepted and tolerated. However, the same vaccine adjuvant formulations applied mucosally may cause damage to delicate epithelia and are likely to be noticeably uncomfortable and therefore much less well tolerated. This is highlighted by recent concerns that use of potent mucosal adjuvants with the capacity to bind gangliosides on the olfactory nerve was associated with neurological side-effects, in particular unilateral facial nerve paralysis, Bell's palsy [25,26]. In addition, it is the necessarily high reactogenicity of these effective vaccine adjuvants at mucosal surfaces that paradoxically may lead to a critical breach in the most efficient barrier to pathogen ingress—the mucosal epithelium.

In this current study we set out to improve mucosal vaccination without compromising the integrity of the mucosae and exposing that surface to opportunistic pathogens. We used the transferrin receptor CD71 as an efficient and natural transporter of its ligand, and were able to demonstrate that a complex macromolecular “cargo” vaccine antigen conjugated to the native transferrin molecule was transported across an intact mucosal epithelium and deposited at the basolateral surface. We selected the HIV gp140 glycoprotein as our model cargo antigen, not because of any inherent ability to elicit potent viral neutralizing antibodies but due to the large size of the trimeric protein conjugate. Successful delivery of such a molecule provides proof of concept for our method and the second or third generation of a designed optimized vaccine antigen could easily replace our current model protein. Targeted transepithelial delivery has previously been described for an insulin–transferrin conjugate that was detected in rat serum 4 hours after oral administration [18]. Crucially, in the experiments described here, we demonstrated that Tf-gp140, which formed high molecular weight complexes (data not shown), retained high affinity binding to CD71. In initial in vitro experiments the transcytosis of Tf-gp140 conjugate, which was abrogated by competition with excess transferrin, occurred with linear accumulation kinetics indicating that the transport was rate-limited or controlled. Specific transport of the conjugate was evident even at very low transferrin concentrations in the apical transwell chamber but was inhibited in the presence of an inhibitor of intracellular transport. These observations together with the demonstration of maintenance of binding kinetics to CD71 indicate that the transport of the transferrin conjugate was mediated by the cognate CD71 receptor. Notably, the Tf-gp140 conjugation process maintained the oligomeric nature of the gp140 that is believed to be important for presentation of the HIV envelope protein in a state that mimics that expressed on the virion [27]. In vivo, appearance of human transferrin in murine blood after application to the vaginal vault or nasal cavity, presumably reflects the high identity between human and murine transferrin proteins and is in agreement with high level expression of CD71 on primary cervico-vaginal epithelium shown here and in other published data on a variety of mucosally associated epithelia including the upper respiratory tract [28–32]. The finding that transcytosed transferrin conjugates localized to cells with dendritic morphology in the underlying stroma is likely due to the expression of CD71 on these cells. These observations suggest that the transferrin component of the conjugate conferred a dual functionality to the conjugate complex of an initial transcytosis of a cargo vaccine antigen across the mucosal epithelial barrier followed by a specific targeting to sub-mucosal APCs.

Our immunogenicity experiments revealed that Tf-gp140, in contrast to gp140 alone, could be highly immunogenic when applied directly to a mucosal surface, eliciting equivalent responses to a standard parenteral prime and mucosal boost regime. This has significant practical potential for vaccine administration as needle based delivery systems have many associated risks, including needle stick injuries and transfer of infections, costs for syringe disposal and the requirement for administration by trained personnel, a particular concern in resource poor settings. Despite demonstrating in vitro and in vivo trafficking of Tf-gp140 across the cervico-vaginal barrier, strikingly, only nasal administration elicited significant antibody responses. There could be a number of explanations for this finding. The vaginal and nasal mucosae are anatomically different. The local environment at the surface of nasal or vaginal mucosae could potentially differ in terms of local secretion of proteases or the resident flora, but it is unlikely the flora could effect such a dramatic result in healthy pathogen free mice [15]. While we clearly observed punctate staining distributed throughout the sub-epithelial lamina propria of both tissues we noted that the nasal epithelia contained much more fluorescent Tf at all timepoints where we looked, though this could be a result of the tissue processing and dissolution of mucus that may trap, and possibly retain, a large proportion of the applied material. Indeed, in vivo imaging of mice revealed that topical application of Tf-Alexa into the vaginal vault maintained a strong fluorescent signal for 24 hours post application (Fig. 3).

A more likely explanation is that the observed differences are due to the immunologic potential of the respective mucosae. The nasopharynx-associated lymphoid tissue (NALT) is immunologically inductive as it contains numerous microfold cells (M cells) and high densities of immune competent cells beneath the follicle-associated epithelium (FAE) while no such follicles exist in the cervico-vaginal epithelium [33–35]. While we observed equally intense CD71 expression on M cells and surrounding epithelium as on non-M cell associated mucosae it is likely that these specialized antigen-sampling cells with their high concentration of immune reactive cells within the follicles play an important role in elicitation of immunity. Within the vagina, the major immune competent cells are dendritic cells and macrophages with the DCs residing in both the submucosal epithelium and in the overlying stratified epithelium and the macrophages being largely restricted to sub-mucosal stroma [14]. DCs located in the stratified epithelium, variously referred to as Langerhans cells (LC), dermal dendritic cells (DDC) or vaginal epithelial dendritic cells (VEDC), are uniquely positioned to capture foreign antigens or pathogens that breach the epithelial barrier as well as perform house-keeping functions such as clearance of apoptotic keratinocytes. However, the ability of these vaginal LC, that are most similar to LC resident in skin, to stimulate naïve T cells is debated and it is reported that these APC populations are at least partly tolerogenic in nature [12,14,36]. The significant differences between the immune responses we observed after topical vaginal or nasal vaccination, even in the context of a systemic priming inoculation, are therefore compatible with the presumed differential inductive potential of the two different mucosae.

Our observation that only the Tf-gp140 conjugate was able to elicit significant immunity after a topical mucosal application while both unconjugated gp140 or the conjugate were equally able to boost a systemic priming vaccination strongly suggests that the quantity of antigen that gets through the mucosal epithelium has a significant impact on the resulting immune response. Indeed the data suggest that there may be a threshold where responses are initiated and amplified but below this threshold immune responses are weak or absent. A dose escalation study will determine if this is correct and will also provide information on the dose-sparing capacity of the Tf-gp140 conjugate. Unlike previous studies involving intranasal immunization [37–42], no adjuvants were used in the present study. It would now be interesting to determine if immunogenicity of the Tf-gp140 conjugate would be enhanced by the use of low concentrations of adjuvant that may be less likely to damage epithelial surfaces. The strikingly enhanced skewing of the serum antibody response toward Th2-type when mice were intranasally immunized with Tf-gp140 indicated that the conjugate may alter antibody-secreting B cell development and differentiation [43–45]. Moreover, the observed increase in the relative proportion of IgA within the murine vaginal vault after Tf-gp140 nasal vaccination suggests that efficient mucosal priming in the absence of exogenous adjuvant may profoundly influence the quality of the local antibody response.

In conclusion, we have demonstrated that a natural endogenous molecular transport system can be utilized to target a large complex antigen, trimeric HIV gp140, to mucosal immune inductive tissue in the absence of adjuvants. Moreover, delivery of antigen using this novel needle-less modality induces a distinctive antibody profile that may be important in generating effective vaccine responses at the portal of pathogen entry.

## Disclosure

The authors declare no conflict of interest.
