# Supplementary material for: Transferrin conjugation confers mucosal molecular targeting to a model HIV-1 trimeric gp140 vaccine antigen
Source: J Control Release. 2012 Mar 10;158(2):240–9. doi: 10.1016/j.jconrel.2011.11.009 (PMC3314955; doi:10.1016/j.jconrel.2011.11.009)
Supplement: Supplementary file 1 — Supplementary material [file mmc1.doc]

**Supplementary Materials and Methods**

***Transferrin – gp140 chemical conjugate***

The Tf-gp140 conjugate and the unconjugated gp140 protein were tested for endotoxin contamination with the limulus amebocyte lysate (LAL) assay as described in the manufacturer’s instructions (Cambrex, USA). Endotoxin content was below the assay detection limit (<0.1 EU/ml).

Successful conjugation was confirmed by protein electrophoresis. Briefly, a 20 μl volume containing 5 μg Tf-gp140 conjugate, gp140 or transferrin was loaded into a 7-10% Novex Tris-Acetate Gel (Invitrogen, UK) and the proteins electrophoresed at 150 V for 45 min. Himark standard (Invitrogen, UK) was used to ascertain relative protein mass.

***Primary endocervical epithelium isolation, flow cytometric analysis and in vitro transwell studies***

Cervical mucosal tissue was collected from pre-menopausal women undergoing therapeutic hysterectomies at St. George's, and Kingston Hospitals (London, United Kingdom) and placed into RPMI. The tissue was dissected to remove as much of the ectocervix and lamina propria as possible, leaving the overlying endocervical epithelia intact. The epithelial tissue was then cut into 0.5 mm^2^ fragments and incubated overnight at 4^o^C in dispase II solution (2.4 U/ml - Roche Diagnostics, Germany) in a 12 well tissue culture plate (Corning, UK). Digested epithelial tissue was further disrupted by mechanical agitation, centrifuged and then incubated with trypsin solution (Sigma, UK) for 30 min to generate a single cell suspension. The primary epithelial cultures were isolated following a protocol by Greenhead et al., (Journal of Virology (2000) 74: 5577- 5586) that is used routinely in our laboratory. This protocol was performed on endocervical explants and the clumps of epithelial cells that were detached from the underlying stroma by dispase II were examined in this previous paper morphologically and with a panel of cytokeratin markers to confirm the cell origin and purity. Fibroblast contamination has not been noted with this protocol. The cells were then centrifuged and re-suspended in keratinocyte serum free medium (KSFM) (Invitrogen, UK). This epithelial cell preparation was used to seed 0.4 μm filter transwell plates (Corning, UK) or tissue culture flasks and placed in a 5% CO_2_ incubator at 37^0^C. Transwells were used for transcytosis studies when tight junction formation was identified (resistivity > 200 Ω/cm^2^) assessed by measuring the transepithelial electrical resistance (TEER) using a Millipore ERS Ohm meter (Millipore, U.S.A). Experiments were initiated when resistivity was greater than 450ohms/cm^2^ and this resistance was maintained throughout the experiment. The KSFM, that contains transferrin, was replaced by Earles balanced salt solution (EBSS) (Sigma, UK) one hour prior to gentle washing with EBSS to remove residual transferrin. 50 μg gp140 or Tf-gp140 with or without the intracellular trafficking inhibitor monensin (Sigma, UK) was then added to the apical chamber. Samples from the basolateral chamber of the transwell were taken at various times and analysed for the presence of transported gp140 or Tf-gp140 by ELISA.

Cells were removed from tissue culture flasks after a rinse with PBS, using non-enzymatic cell dissociation solution (Sigma) to prevent cleavage of cell surface receptors, counted and resuspended at 1 x 10^7^ cells/ml in FACs buffer (1% BSA / PBS). 100 μl of prepared cells were incubated with 10 μl of a 4 mg/ml concentration of unlabelled mouse anti-human CD71 mAb (Abcam, UK) for 1 hour at 4^o^C. The cells were washed twice in 1 ml FACs buffer, resuspended in residual medium, approximately 100 μl, before addition of 10 μl of PE-labeled goat anti-mouse (Fab’)_2_-specific antibody (Dako, UK) diluted 1:15 in FACs buffer. The cells were incubated for 1 hour at 4^o^C before being washed in PBS and fixed in 1.5% formaldehyde. Stained cells were quantified using a Beckman Coulter FC500.

***ELISA***

***Anti-human transferrin ELISA*:** A commercially available human transferrin-specific ELISA was used as per the manufacturer’s instruction (Universal Biologicals (Cambridge) Ltd., UK).

***CN54gp140 antigen-specific ELISA*:** Briefly, 50 μl of the Lectin *Galanthus nivalis* (Sigma, UK) dissolved in carbonate buffer was used to coat high binding Nunc Maxisorp plates (Corning, UK), at 5 μg/ml overnight at 4^o^C. The plates were washed three times with PBS containing 0.05% Tween 20 (PBS-T) before blocking with 200 μl 1% BSA (Sigma, UK). Undiluted samples (50 μl/well) or a gp140 standard (range 100 ng/ml – 0.78125 ng/ml; 2-fold dilution series) were added to the ELISA plate and were incubated for one hour then washed four times in PBS-T before the addition of 50 μl/well of 1 μg/ml anti-gp41 human monoclonal antibody 5F3 (Polymun, Austria) diluted in 1% BSA [38]. Plates were incubated for 1 hour and washed four times in PBS-T. Goat anti-human-IgG (γ-chain) conjugated with horseradish peroxidase (HRP; Zymed, Germany) diluted 1:5000 in 1% BSA was then added to each well and incubated for 1 hour. Plates were washed four times with PBS-T before developing with 100 μl/well o-phenylenediamine dihydrochloride (OPD; Sigma, UK), and the reaction stopped with 100 μl/well 1 M H_2_SO_4_. Absorbance was read at 492 nm on a KC4 spectrophotometer (BioTek, UK).

***Anti-CN54 antibody ELISA*:** Antigen-specific gp140 binding antibodies against recombinant CN54gp140 were measured using a standardized ELISA. Maxisorp high binding 96-well plates were coated with 50 μl CN54 gp140 at 5 μg/ml in PBS for 1 hour at 37°C. Plates were washed three times in PBS-T before blocking with 200 μl PBS-T containing 10% foetal bovine serum for 1 hour at 37°C. After further washing, sera diluted 1/100 or mucosal wash samples diluted 1/10 were added at 50 μl /well and incubated for 1 hour at 37°C. Plates were washed four times before the addition of 50 μl of either a 1/10,000 dilution of goat anti-mouse IgG-HRP, a 1/1000 dilution goat anti-mouse IgG2a-HRP, a 1/5000 dilution goat anti-mouse IgG1-HRP or a 1/2000 dilution of biotinylated anti-mouse IgA (all Southern Biotech, UK) secondary antibody and incubated for 1 hour at 37°C. The plates were washed four times and developed with 50 μl/well of KPL SureBlue TMB substrate (Insight Biotechnology, UK). The IgA required a further Streptavidin-HRP (R&D systems) amplification step prior to TMB development. The reaction was stopped after 15min by adding 50 μl/well 1 M H_2_SO_4_, and the absorbance read at 450 nm on a KC4 spectrophotometer.

***Immunohistochemistry***

To visualise the ability of transferrin or the Tf-gp140 conjugate to translocate into the submucosal environment from an external luminal compartment, medroxy-progesterone treated mice received a topical intravaginal or intranasal 20 μl volume of 100 μg Tf-Alexa 647 (Tf-Alexa; Invitrogen, UK). For the intravaginal examinations, mice were killed two hours after Tf-Alexa application and their vaginas placed into 500 μl PBS. The vaginal tissue was trimmed to remove any fatty deposits before being embedded in OCT Cryomatrix (RA Lamb, USA) and flash frozen in liquid nitrogen. After intranasal application of Tf-Alexa, the mice were killed after 45 minutes, their heads were removed from the carcasses and nasal tissue isolated by the removal of skin, the lower jaw, and eyes. Nasal tissue was then fixed in methanol-free formaldehyde and incubated overnight at 4^o^C. Fixation was followed by 7 days of decalcification in 3 changes of 10% EDTA. Before embedding in OCT matrix, tissue samples were placed into 10% sucrose/PBS for 2 hrs and then placed into 30% sucrose/PBS for a further 16 hours. Samples were cut into 6-8 μm sections using a Cryotome (Bright laboratories, UK.), placed on a glass slide and then stored in foil at –20^o^C until further processing. Sections were allowed to defrost at room temperature before fixation in an acetone/methanol mixture (1:1, v/v) for 10 minutes. Samples were then washed briefly 3 times in PBS (Gibco, UK) before mounting in mounting media containing DAPI (Vector Laboratories Ltd., UK).

To stain for MHC class II antigen, slides were incubated in TNB blocking buffer (NEN Life Sciences, USA) as per the manufacturer’s instructions. Endogenous biotin and avidin activity was quenched using the avidin-biotin block (Vector Laboratories) while endogenous peroxidase activity was quenched using 3% (v/v) H_2_O_2_. Sections were then incubated with 50 µl of 2 µg/ml anti-MHC class II antibody (BD Pharmingen; clone - M5/114.15.2) in PBS-T for 1 hour at room temperature before washing in PBS, incubation for 30 min at room temperature with a 1/1000 dilution of biotinylated goat anti-rat antibody (KPL, USA) diluted in TNB buffer, washing for 10 min before addition of a 1:100 dilution of Streptavidin-HRP (R&D systems) in PBS for 30 min. Slides were again washed for 10 min and then Tyramide Signal Amplification (TSA) reagent (Perkin Elmer, UK) diluted 1:50 in PBS was added to the sections for 10 min. Slides were washed in PBS and mounted as described earlier. To visualise gp140 within vaginal tissues, 100 ul of a 2 ug/ml solution of 5F3 (Polymun, Austria) was used as the primary antibody, while goat anti-human-IgG (γ-chain) conjugated with horseradish peroxidase (HRP; Zymed, Germany) was used at a 1:500 dilution as the secondary antibody. Tyramide amplification was carried out as above.

Stained slides were visualized using a Leica DMI6000B microscope with a DFC320 camera at x400 magnification.

Endocervical tissue from patients undergoing planned therapeutic hysterectomy (local Research Ethics Committee approval was obtained) were cut into 3mm^3^ samples and placed into 10% formaldehyde overnight at 4^o^C. Tissue samples were loaded into histocasettes (Fisher, UK) and paraffin embedded overnight. The following day, 6 μm sections were cut using a microtome and processed for Immunohistochemical analysis by sequential exposure to three five-minute washes in xylene, two five-minute washes in absolute ethanol, two five-minute washes in 95% ethanol, and one five-minute wash in PBS. Slides were immersed into 10 mM citric acid pH6 and warmed in a microwave. Slides were allowed to cool for 20 minutes at room temperature before being rinsed twice in deionized water. Endogenous peroxidase activity was quenched using H_2_O_2_ for five minutes before rinsing for five minutes in PBS. Sections were then stained using the Super Sensitive Link-labelled detection system (Biogenex, UK). Briefly, Power Block™ was used to block slides while a 1/100 dilution of anti-CD71 primary antibody (clone H68.1) was used to detect transferrin receptor. Slides were then incubated with Multilink as per the manufacturers instructions before developing with DAB and counterstaining with hematoxylin. Images were recorded and photographed as described above.
